# Clinical Importance of the Persistent Primitive Trigeminal Artery in Vascular Lesions and Its Role in Endovascular Treatment

**DOI:** 10.3389/fneur.2022.928608

**Published:** 2022-07-11

**Authors:** Yiheng Wang, Jinlu Yu

**Affiliations:** Department of Neurosurgery, The First Hospital of Jilin University, Changchun, China

**Keywords:** persistent primitive trigeminal artery, endovascular treatment, vascular lesion, clinical importance, review

## Abstract

The persistent primitive trigeminal artery (PPTA) extends from the internal carotid artery to the basilar artery between the origins of the anterior inferior cerebellar artery and superior cerebellar artery. PPTAs have complex anatomical characteristics. Salas and Saltzman classifications are most often used in PPTAs. The PPTA can play many roles in vascular lesions, including intracranial aneurysms, brain arteriovenous malformations, trigeminal artery-cavernous fistulas, Moyamoya disease, and large vessel occlusion. For these lesions, surgical treatment is difficult due to the deep location and complex anatomy of the PPTA, but endovascular treatment (EVT) has emerged as a good alternative. Currently, a complete review of the clinical importance of the PPTA in terms of its role in the development and EVT of vascular lesions is lacking. Therefore, we conducted a PubMed search, performed a review of the relevant extracted literature and cataloged our experience with PPTAs. By review, we found that a thorough understanding of the anatomical and angiographic features of this PPTA is of utmost importance when making therapeutic decisions for any of these pathological conditions.

## Introduction

According to the Padget description, in an embryo, there are four channels between the carotid and vertebrobasilar arteries, namely, the trigeminal, otic, hypoglossal, and proatalantal intersegmental arteries (1). During typical embryonic development, these channels eventually regress, but occasionally, they persist, with the persistent primitive trigeminal artery (PPTA) being the most common of the persistent carotid-basilar connections, having a reported prevalence of 0.061–0.6% and accounting for 80–85% of persistent arteries (2–4).

**Figure 1:**
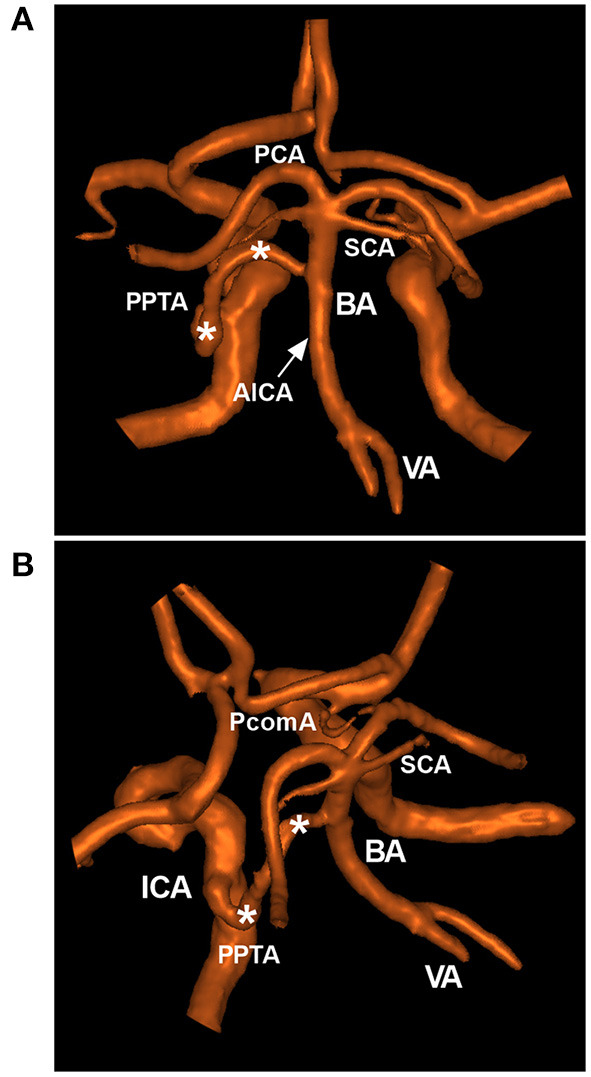
Typical PPTA in MRA. **(A,B)**: Posterior anterior **(A)** and superior oblique MRA views **(B)** showing a typical PPTA (asterisks) from the cavernous ICA to the BA between the SCA and AICA. The arrow **(A)** indicates the origin of the AICA. AICA, anterior inferior cerebellar artery; BA, basilar artery; ICA, internal carotid artery; MRA, magnetic resonance angiography; PCA, posterior cerebral artery; PcomA, posterior communicating artery; PPTA, persistent primitive trigeminal artery; SCA, superior cerebellar artery; VA, vertebral artery.

The PPTA is an important and complex artery (Figure 1). It has been associated with a variety of vascular lesions, including intracranial aneurysm, brain arteriovenous malformation (BAVM), trigeminal artery-cavernous fistula (TCF), Moyamoya disease (MMD), and acute and chronic large vessel occlusion (5, 6). Therefore, a thorough understanding of the anatomical and angiographic features of the PPTA is of utmost importance when making therapeutic decisions for any of these pathological conditions.

Surgical treatment of vascular lesions of the PPTA is difficult due to its deep location and complex anatomy, but endovascular treatment (EVT) has emerged as a good alternative (7). Currently, a complete review of the clinical importance of the PPTA in terms of its role in the development and EVT of vascular lesions is lacking. Therefore, we felt it necessary to conduct a review of the literature from a PubMed search and to recount our experience with treating vascular lesions of the PPTA. Additionally, we provide important images and educational cases in this review to increase reading interest.

## Angiographic Characteristics

The PPTA extends from the internal carotid artery (ICA) to the basilar artery (BA) between the origins of the anterior inferior cerebellar artery (AICA) and superior cerebellar artery (SCA) (4). Most (85%) PPTAs originate from the posterior wall of the cavernous segment of the ICA, and few (15%) originate from the petrous segment (8). The PPTA usually arises by itself from the ICA or has a common origin with the meningohypophyseal trunk (9). Except for the collateral channel between the anterior and posterior circulations, the PPTA trunk can sprout pontine perforating arteries and branch to the trigeminal ganglion (10, 11).

**Figure 2:**
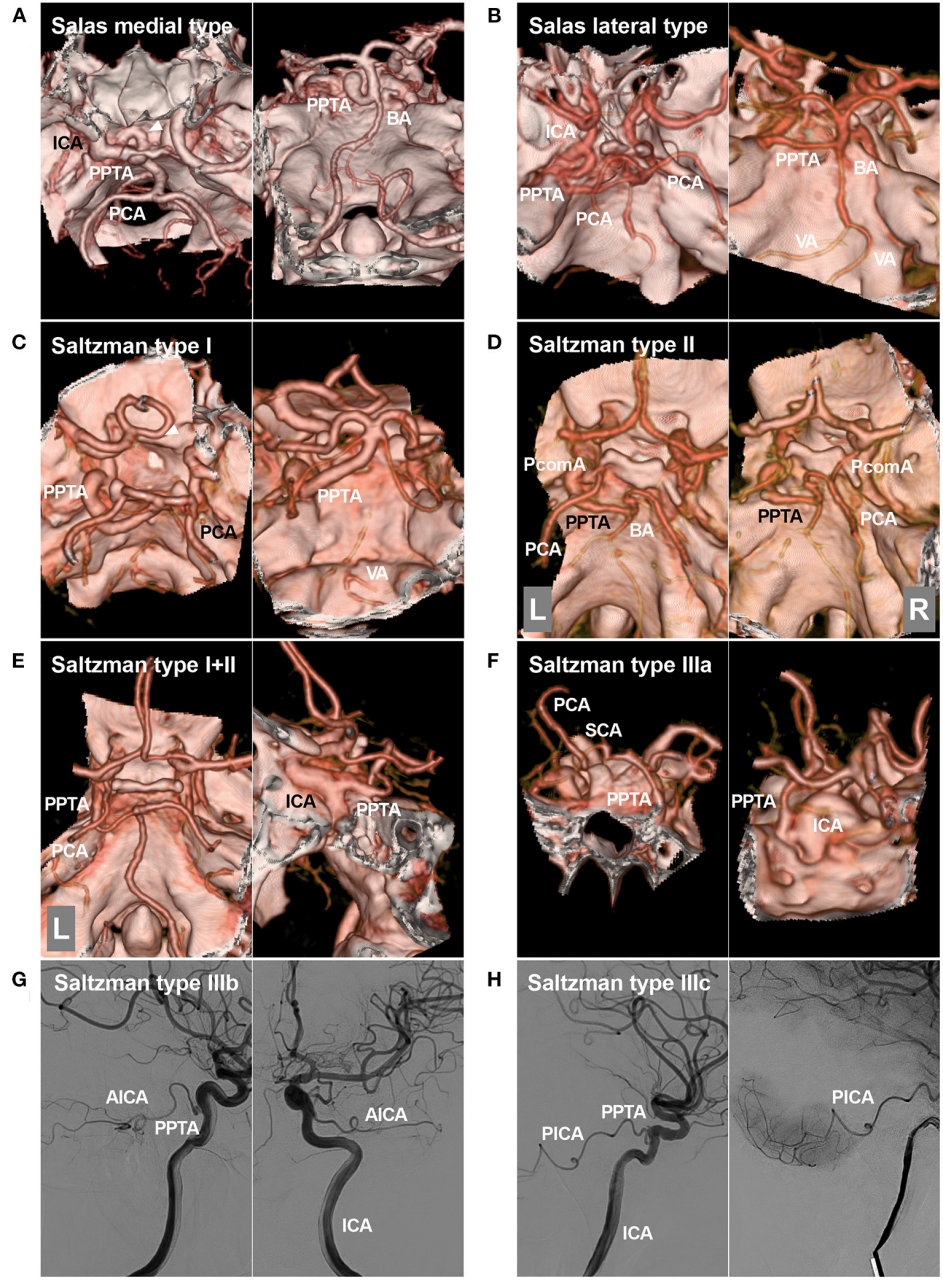
Salas and Saltzman classifications. **(A)**: Salas medial (sphenoidal) type. Left, CTA showing the PPTA arising from the ICA, running medially into the sella (arrowhead), and then crossing the dorsum sellae. Right: CTA showing the PPTA connecting to the BA. **(B)**: Salas lateral (petrosal) type. Left, CTA showing the PPTA arising from the ICA, running laterally, and then crossing the cavernous sinus. Right: CTA showing the PPTA connecting with the BA. **(C)**: Saltzman type I. Left, CTA showing the PPTA arising from the ICA. Right: CTA showing the PPTA connecting with the BA. In this case, the PPTA is strong and hyperplastic, and the PcomAs and vertebrobasilar artery are hypoplastic. **(D)**: Saltzman type II. Left, CTA showing the left PPTA and the left fetal-type PCA; Right, CTA showing the right PCA receiving its blood supply from a patent PcomA. The proximal BA is well developed. **(E)**: Saltzman combined type (I+II). Left, CTA showing that the left PCA is fetal-type and not connected with the BA, the right PCA originates from the BA, and no PcomA can be seen; Right: CTA showing that the PPTA arises from the left ICA. The vertebrobasilar arteries are normally developed. **(F)**: CTA showing a type IIIa PPTA arising from the ICA and then extending to the opposite SCA. **(G)**: DSA of the ICA showing a type IIIb PPTA arising from the ICA and then extending into the AICA. **(H)**: DSA of the ICA showing a type IIIc PPTA arising from the ICA and then extending into the PICA. AICA, anterior inferior cerebellar artery; BA, basilar artery; CTA, computed tomography angiography; DSA, digital subtraction angiography; ICA, internal carotid artery; L, left; PCA, posterior cerebral artery; PcomA, posterior communicating artery; PICA, posterior inferior cerebellar artery; PPTA, persistent primitive trigeminal artery; R, right; SCA, superior cerebellar artery; VA, vertebral artery.

Based on the course in angiography, Salas et al. (10) classified PPTAs into medial (sphenoidal) and lateral (petrosal) types; the medial type reflects the direct perforation of the central portion of the dorsum sellae and forms an anastomosis with the BA, while the lateral type courses along the lateral portion of the dorsum sellae, turns toward the central side, and then forms an anastomosis with the BA (Figures 2A,B) (12, 13).

Except for the Salas classification of the PPTA, the most common is the modified Saltzman classification (types I, II, and III) (10, 14–17). Saltzman type I PPTA enters the BA between the SCA and AICA; the PPTA supplies the BA, SCAs, and posterior cerebral arteries (PCAs); the posterior communicating arteries (PcomAs), vertebral artery (VA) and proximal BA may be absent or hypoplastic, and this hypoplasia should not be confused with stenosis (Figure 2C). In type II, the PPTA supplies the BA and SCAs, the proximal BA is well formed, and PcomAs are present and, with the BA, contribute to the distal posterior circulation (Figure 2D). In the combined type (I+II), the PPTA supplies the BA and bilateral SCA as well as the opposite or ipsilateral PCA, while the other PCA is supplied by a patent PcomA (Figure 2E). In type III, the PPTA terminates as a cerebellar artery [specifically, types IIIa, b, and c PPTAs terminate as the SCA, AICA, and posterior inferior cerebellar artery (PICA), respectively] (Figures 2F–H) (18, 19).

In the embryonic vascular system, trigeminal, stapedial, and ophthalmic arteries can develop at a specific stage (20–23). Therefore, PPTA can incorporate the primitive stapedial and ophthalmic arteries, resulting in complex variants (24). One is the stapedo-trigeminal variant, in which the PPTA takes over the territory of the intracranial branch of the primitive stapedial artery, presenting with the middle or accessory meningeal artery originating from the BA (20). Another is the ophthalmo-stapedo-trigeminal variant, in which the ophthalmic artery originates from the BA (25). The other is the stapedo-trigeminal-cerebellar variant, in which the middle or accessory meningeal artery supplies the cerebellum (21).

## Role of the PPTA in Aneurysms

The PPTA plays two roles in the treatment of aneurysms: one is as a path for delivering coils to distal aneurysms of the posterior circulation, which is not discussed here (26). The other is as the parent artery itself of the aneurysms, which occur in 14–32% of PPTAs (27–29). Various etiologies for aneurysms arising from the PPTA have been suggested, including dysplasia of the PPTA wall and hemodynamic stress on the PPTA (30).

**Figure 3:**
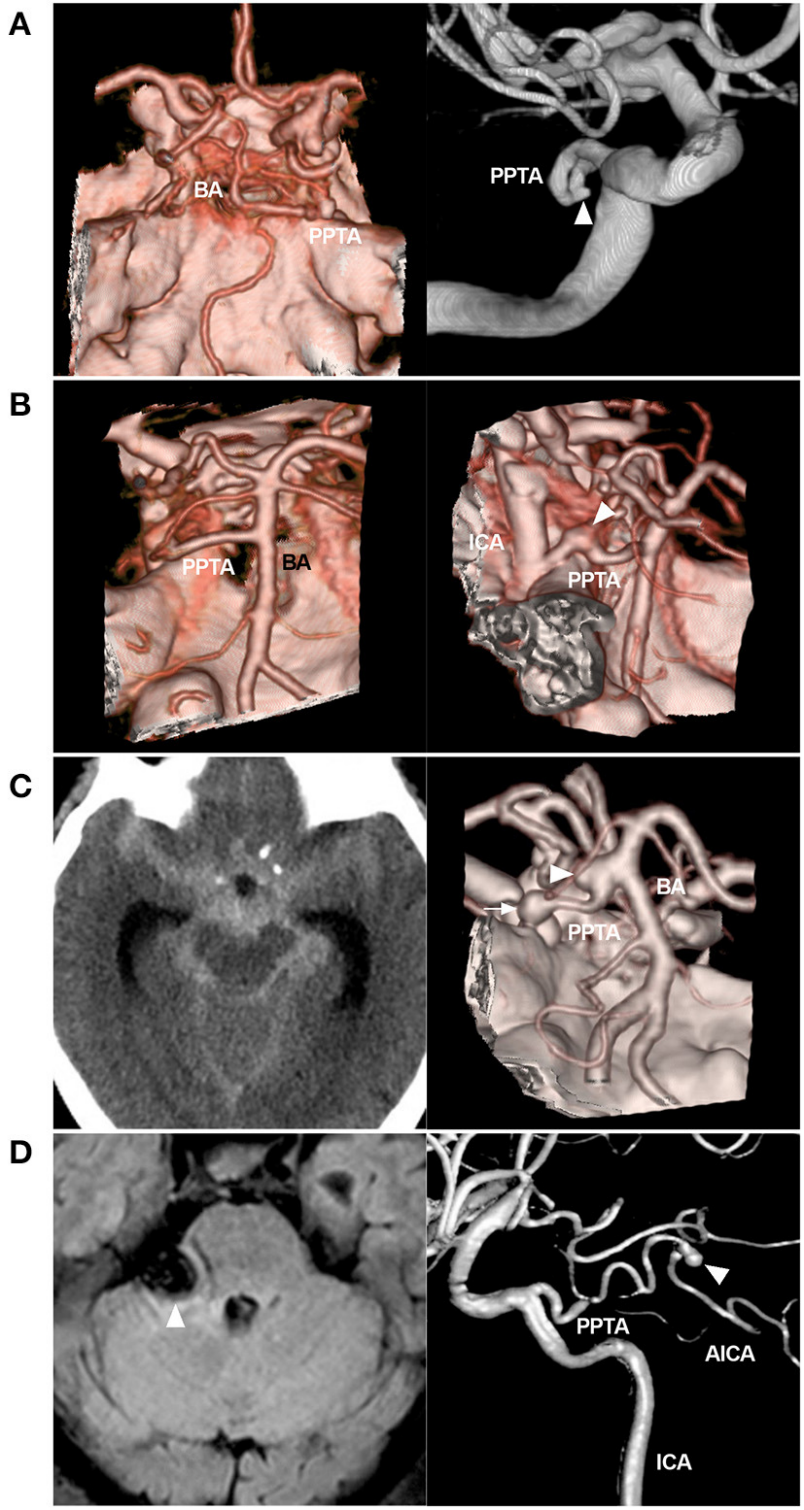
Variant aneurysms of the PPTA. **(A)**: Left: CTA showing the PPTA; Right: DSA of the ICA showing a small aneurysm (arrowhead) on the middle PPTA trunk. **(B)**: Left: CTA showing the PPTA; Right: CTA showing a fusiform aneurysm (arrowhead) on the PPTA trunk near the origin. **(C)**: Left: CT showing a subarachnoid hemorrhage; Right: CTA showing two aneurysms, one (arrow) located on the PPTA trunk and the other (arrowhead) at the junction of the PPTA and BA. **(D)**: Left: MRI showing a mass (arrowhead) in the cerebellopontine angle region, compressing the brainstem; Right: Three-dimensional DSA showing a Saltzman type IIIb PPTA, which continues to the AICA; a distal aneurysm of the AICA can be seen (arrowhead). AICA, anterior inferior cerebellar artery; BA, basilar artery; CT, computed tomography; CTA, computed tomography angiography; DSA, digital subtraction angiography; ICA, internal carotid artery; MRI, magnetic resonance imaging; PPTA, persistent primitive trigeminal artery.

These aneurysms can be divided into ruptured or unruptured, saccular or fusiform (dissection), small or large, and single or multiple aneurysms (Figure 3). PPTA aneurysms can grow along the course of the vessel, including the PPTA-ICA junction, its trunk in the cavernous sinus or cistern, and the PPTA-BA junction (2, 31). Saccular aneurysms are common, and most of them are located at the PPTA-ICA junction (32).

**Figure 4:**
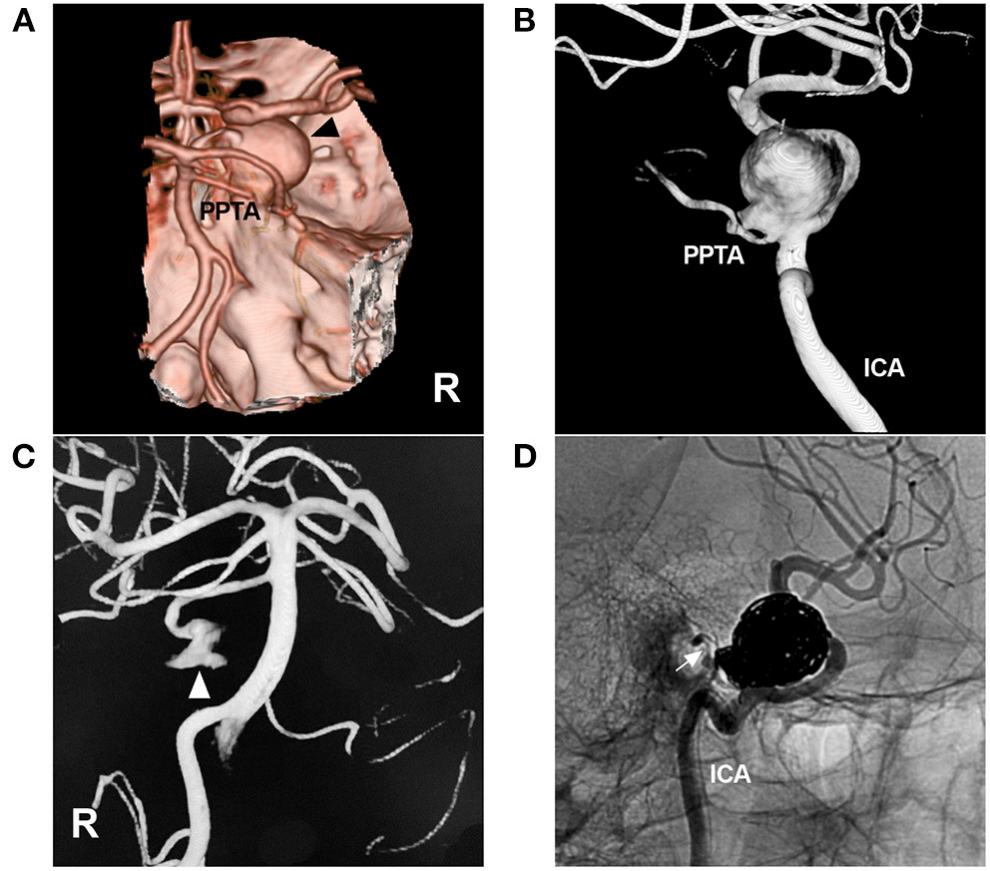
Coiling of an aneurysm of the PPTA-ICA junction. **(A)**: CTA showing a large right parasellar aneurysm (arrowhead); a PPTA can be seen. **(B)**: Three-dimensional DSA of the ICA showing the aneurysm located at the junction of the PPTA and ICA. **(C)**: Three-dimensional DSA of the right VA showing part of the PPTA aneurysm (arrowhead). **(D)**: Unsubtracted DSA showing the aneurysm coiled via the ICA, preserving the PPTA (arrow). CTA, computed tomography angiography; DSA, digital subtraction angiography; ICA, internal carotid artery; PPTA, persistent primitive trigeminal artery; R, right; VA, vertebral artery.

Ruptured aneurysms are typically associated with PPTA-cavernous fistulas (discussed later) or subarachnoid hemorrhage. Large or giant unruptured aneurysms can result in cranial nerve palsies or trigeminal neuralgia due to mass effects (33). EVT is a good option for treating these ruptured or symptomatic aneurysms, including reconstructive or deconstructive techniques. Selective coiling with or without balloon/stent assistance can be used to reconstruct PPTA with bifurcation aneurysm or saccular dissection (Figure 4) (31, 34).

If the aneurysm is fusiform or incorporates an excessively large proportion of the PPTA wall, reconstructive techniques can be difficult, and parent artery occlusion (PAO) can be used (33). It is important to evaluate the hemodynamic balance between the PPTA and vertebrobasilar system; if the vertebrobasilar system is well developed and PcomAs are present, PAO of the PPTA can be performed safely.

In Saltzman type I PPTA aneurysms, the blood flow of the posterior circulation from the proximal BA and VA may be insufficient or uncertain. Therefore, a preprocedure balloon occlusion test (BOT) is important for evaluating whether the proximal BA has anterograde flow from the VAs to maintain satisfactory perfusion to the distal vasculature after PAO of PPTA aneurysms. During the BOT, the balloon should be inflated into the PPTA; however, when the PPTA is thin or it is difficult for the balloon to go into the PPTA, it can be placed in the ICA covering the PPTA origin. The BOT should be performed when the patient is awake to allow for real-time evaluation of neurological deficits. Additionally, mean arterial pressure can be reduced to 70% of baseline during the BOT to evaluate neurological deficits (35).

In patients who cannot tolerate the BOT, strong consideration should be given to EVT allowing for PPTA preservation (36). However, even if patients can tolerate the BOT, after PAO, there remains the risk of pons and midbrain infarction or trigeminal nerve ischemia because PAO of the PPTA trunk can sacrifice some aberrant perforating branches arising from the PPTA itself (37, 38). In lateral-type PPTAs in particular, *in situ* thrombosis-associated infarction is higher than in the medial type, as Salas et al. (10) showed that brainstem perforators often arise from the PPTA with lateral cisternal courses.

PAO for PPTA aneurysms can be performed by coiling the PPTA together with an aneurysm with/without the assistance of a traditional stent or flow diverting stent (FDS) in the ICA or BA to protect or reconstruct the ICA or BA and even covered stent implantation in the ICA (39, 40). For Saltzman type IIIa-c PPTA aneurysms, it can be difficult to preserve the cerebellar arteries, and the occlusion of the proximal segments can result in brainstem infarction (41, 42). In addition, in type IIIb AICA aneurysms, when PAO is planned, the internal auditory artery may be occluded (43). The risks of these procedures must be considered.

## Trigemino Artery-Cavernous Fistulas

TCF is a rare high-flow fistula between a PPTA and cavernous sinus (CS), following either spontaneous rupture of an aneurysm at the ICA-PPTA junction and PPTA trunks or traumatic tearing of the PPTA (44, 45). Most TCFs require intervention, especially when there is progressive vision loss and cortical and/or deep venous drainage (30). EVT can bring a good prognosis. In Miller et al.'s (46) review, overall, the rate of neurological deficit following EVT of a TCF was only 7%, and there was no mortality. Currently, EVT can be the first-line option for TCFs, including transarterial, transvenous, and combined transarterial and transvenous packing of the CS with coils or intra-aneurysmal coiling (47–49). When coiling the CS, liquid embolic agents can be used as a supplement (50, 51).

For EVT, it is important to determine the type of PPTA and treat it accordingly and to ascertain whether it can be occluded. For TCFs of Saltzman type I PPTAs with a hypoplastic vertebrobasilar system or Saltzman type III PPTAs, the PPTA should be preserved (50, 52). During EVT, intra-aneurysmal coiling for treating TCF while preserving the PPTA is not difficult because the aneurysm may have a narrow neck (53). Sometimes, balloon or stent-assisted coiling can be used (30). In addition, packing the CS from both the ICA and BA with a double catheter system can avoid coil migration by simultaneous control of the bidirectional flows to preserve PPTA (54). Detachable balloon embolization is often difficult because of the tortuous nature of the PPTA and for small fistulas, and it is rarely used (55).

For TCFs without aneurysm necks or with tortuous PPTA, a transvenous approach *via* an accessible inferior petrosal sinus route may be suitable (53). Because transvenous packing of the CS requires a large volume of coils, ocular symptom exacerbation and venous infarction may occur, but these can usually be avoided by coiling the venous pouch of the TCF (30). PAO of the PPTA can also heal a TCF; however, if the PPTA has a high flow, PAO with coiling is often difficult (56, 57). In such cases, coiling-in bridging stents may be useful for performing PAO for the PPTA (58). Due to its diversion property, an FDS may be used as a treatment for TCFs. In a report by Yoon et al. (59), traumatic TCF was treated with an FDS in the ICA and 1 coil in the CS, resulting in complete fistula obliteration despite undercoiling of the CS, which indicated that TCF can regress after reducing blood flow.

## Role in Acute Large Vessel Occlusion

**Figure 5:**
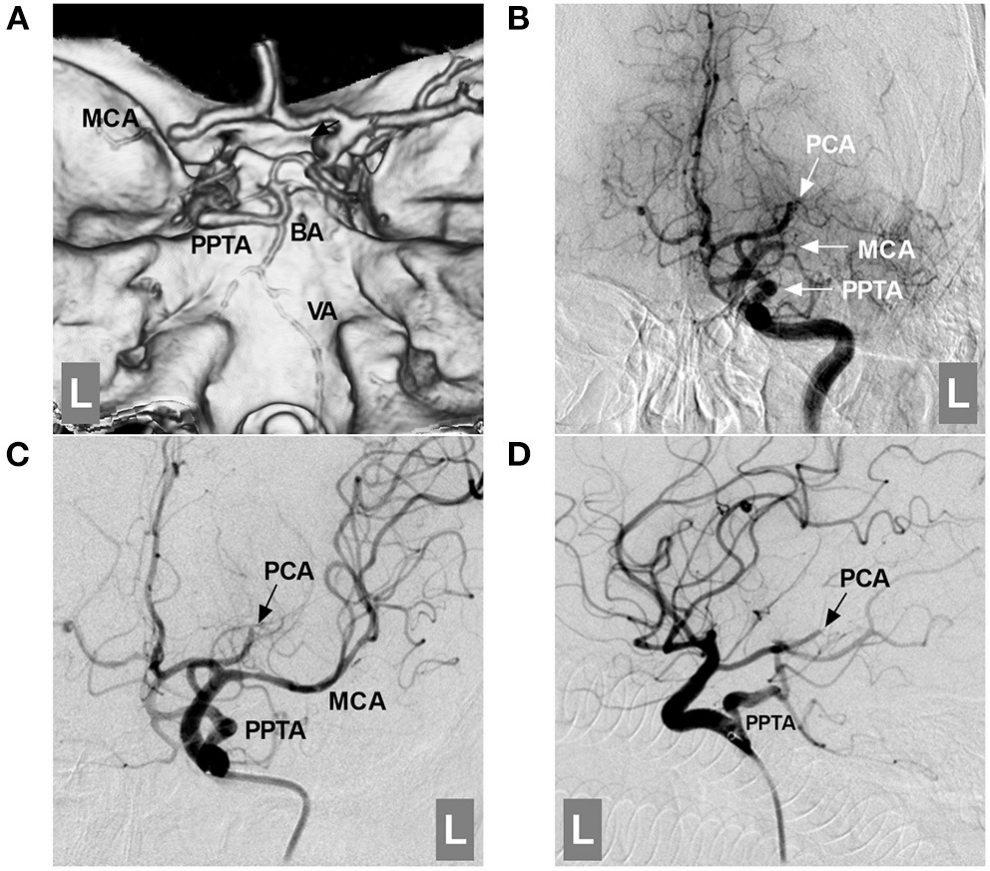
Role of the PPTA in acute large vessel occlusion. **(A)**: CTA showing an occluded left MCA and a PPTA connected with the BA; the proximal BA and VA were hypoplastic. **(B)**: DSA of the ICA showing an occluded left MCA and PCA (arrows with MCA and PCA); the PPTA (arrow with PPTA) is unaffected. **(C,D)**: DSA of the ICA showing that the MCA was revascularized after mechanical thrombectomy; the PCA was fetal-type and occluded distally (arrows with PCA). BA, basilar artery; CTA, computed tomography angiography; DSA, digital subtraction angiography; ICA, internal carotid artery; L, left; MCA, middle cerebral artery; PCA, posterior cerebral artery; PPTA, persistent primitive trigeminal artery; VA, vertebral artery.

Large vessel occlusion is often the result of a high load of cardiogenic embolus. Hypoplastic PPTAs are often spared from large vessel occlusion (Figure 5). However, the same cannot be said for hyperplastic PPTAs; the occlusions may be very extensive and involve the ICA, PPTA, BA, PCA, SCA, and even the anterior and posterior circulations (3, 60–62). Acute large vessel occlusion is life-threatening (63). Currently, mechanical thrombectomy (MT) can achieve a high rate of recanalization with a low rate of complications (64).

In MT for cases with PPTA, the posterior circulation approach is often difficult because 42.5% of PPTAs often have a hypoplastic BA, and MT must be performed *via* the PPTA (17, 65). In a report by Imahori et al. (62), a patient suffered acute occlusions of the middle cerebral artery (MCA) and BA; MCA occlusion was given conversative treatment, BA occlusion was successfully treated by MT *via* the PPTA, and the patient survived after MT. In Horio et al.'s (65) report, a patient with acute occlusions of the ICA and PPTA was treated with MT, and a good recovery was obtained. Therefore, clinicians should be aware that the PPTA can be involved in acute large vessel occlusion (62). The PPTA can be used as an access route for MT.

## Role in MMD and ICA Stenosis/Occlusion

### Moyamoya Disease

**Figure 6:**
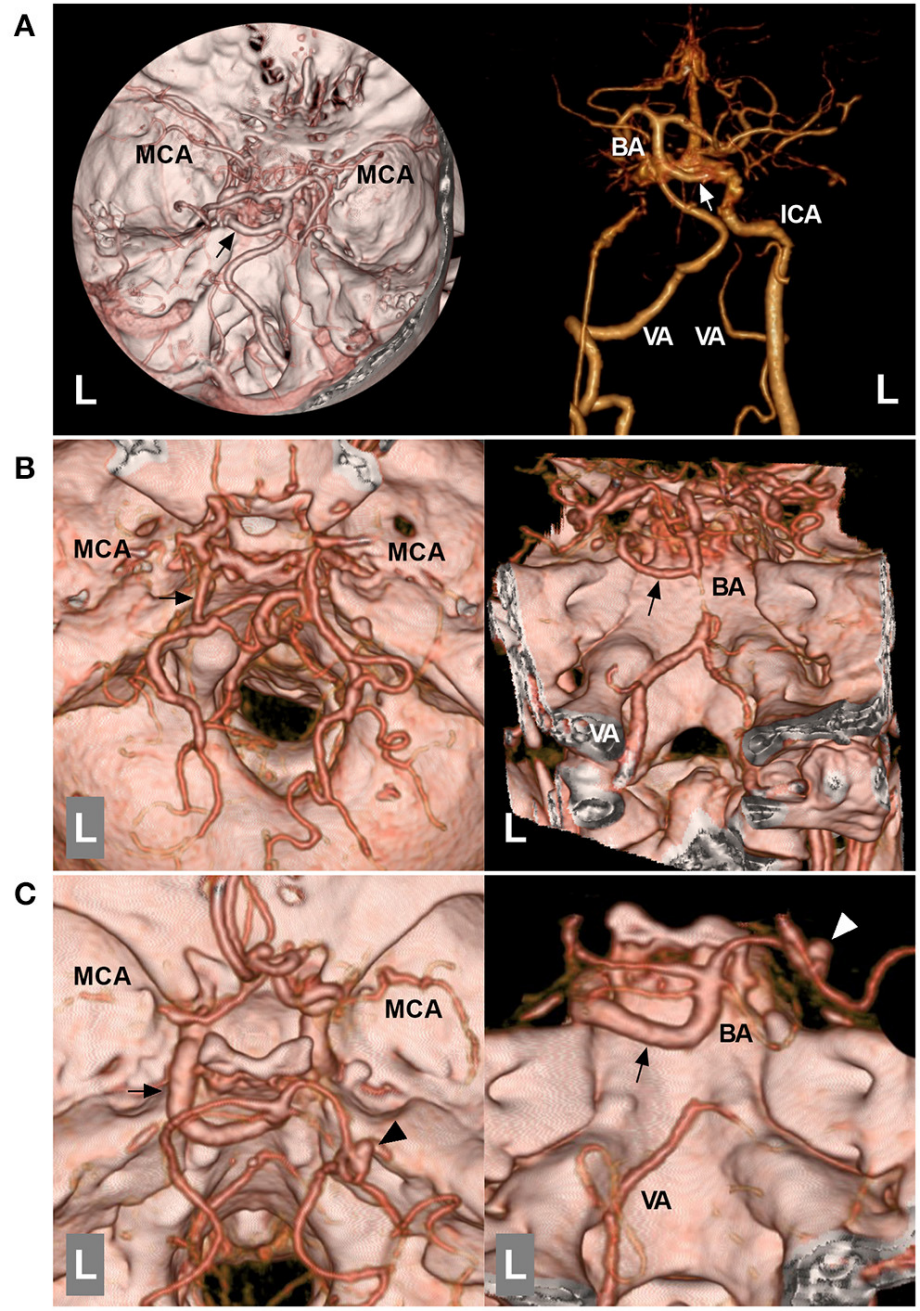
Role of the PPTA in MMD. **(A)**: Left: CTA of the superior inferior view showing that the bilateral MCAs have partially disappeared, and the left PPTA (arrow) is indicated. Right: CTA with bone removal showing that the distal ICA termination is occluded, and the left ICA is connected with the PPTA (arrow). **(B)**: Left: CTA of superior inferior view showing that the bilateral MCAs are obscured, and the left PPTA (arrow) is indicated; Right: CTA of posterior anterior view showing that the proximal BA was hypoplastic, and the left PPTA (arrow) is indicated. **(C)**: Left: CTA of superior inferior view showing that the bilateral MCAs have partially disappeared, and a left PPTA (arrow) and an aneurysm (arrowhead) on the right PCA can be seen; Right: CTA of posterior anterior view showing a hypoplastic proximal BA and an aneurysm (arrowhead) on the right PCA, and the left PPTA (arrow) is indicated. BA, basilar artery; CTA, computed tomography angiography; ICA, internal carotid artery; L, left; MCA, middle cerebral artery; MMD, Moyamoya disease; PCA, posterior cerebral artery; PPTA, persistent primitive trigeminal artery; VA, vertebral artery.

Coexistence of PPTA with MMD has been reported (Figure 6) (66, 67). Congenital factors might be involved in the coexistence of PPTA with MMD because the period during which the PPTA disappears at the embryonic stage (5–14 mm) almost corresponds to the period (11.5–14 mm) in which the vascular state is similar to the Moyamoya phenomena (67–70). In a report by Uchino et al., the incidence of PPTA was ten times higher in MMD, which supported the role of congenital factors (71). However, some evidence suggests that the presence of a PPTA is associated with MMD only incidentally (72, 73).

The PPTA plays an important role in MMD; it is often associated with vertebrobasilar hypoplasia, and the posterior circulation in MMD is highly dependent on the PPTA (74). When the ICA is occluded, the PPTA may become the only blood supply to the whole brain (75). Therefore, if the PPTA stenoses, aggressive transluminal angioplasty is recommended. In MMD, the upper BA, SCA and PCA may be associated with aneurysms (35). The PPTA can be used as the path for endovascular coiling (76).

### Chronic ICA Stenosis/Occlusion

In cervical ICA stenosis/occlusion, the PPTA may be either beneficial or detrimental. ICA stenosis below the PPTA may be a potential source for microemboli because artery-to-artery embolism in the BA territory may occur *via* the PPTA with anterograde flow (77, 78); in this case, the PPTA is detrimental. Carotid angioplasty can be a possible therapeutic option (77, 79). In severe stenosis or occlusion of the cervical ICA, the blood flow of the PPTA can be reversed, through which the ICA territory will be supplied by the posterior circulation (80–83); in this case, although the PPTA is beneficial, carotid angioplasty may be recommended to increase brain perfusion (84). In Foerch et al.'s (85) report, a patient presented with severe stenosis of the cervical ICA, and the PPTA had a retrograde flow from the posterior circulation to the ipsilateral ICA territory; after carotid angioplasty to restore normal ICA flow, PPTA orthograde filling was visible. In extremely rare cases, due to the absence of the proximal ICA, reversed hemodynamic stress can result in a PPTA-BA aneurysm that must be treated (86).

## Role in BAVMS

**Figure 7:**
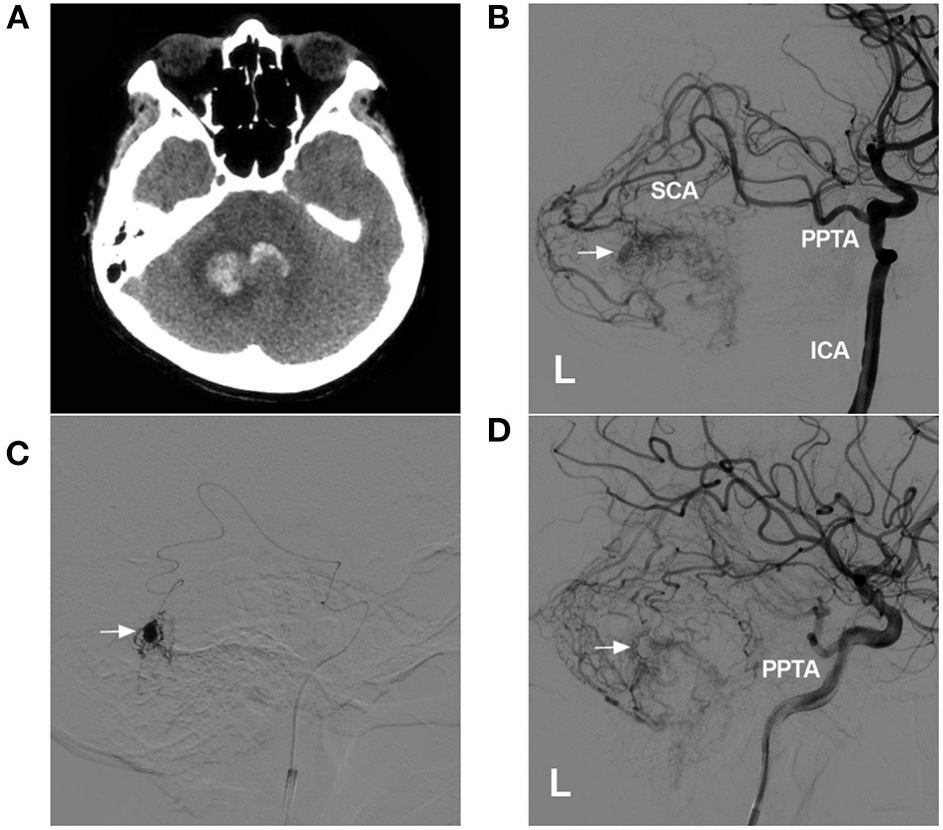
Role of the PPTA as a transarterial path for a BAVM. **(A)**: CTA showing hemorrhage of the cerebellar vermis and fourth ventricle. **(B)**: DSA of the left ICA showing a BAVM supplied by a PPTA via the SCA. An intranidal aneurysm is indicated (arrow). **(C)**: Superselective angiogram of the microcatheter showing the aneurysm (arrow). **(D)**: Postoperative DSA of the left ICA showing that the intranidal aneurysm had been embolized (arrow). BAVM, brain arteriovenous malformation; CT, computed tomography; CTA, computed tomography angiography; DSA, digital subtraction angiography; ICA, internal carotid artery; L, left; SCA, superior cerebellar artery; PPTA, persistent primitive trigeminal artery.

Coincidence of PPTA with BAVMs is rare, with an incidence of only 4.5% (28). When BAVMs are associated with the PPTA, they are often located in the cerebellum, occipital lobe or intrinsic trigeminal nerve (87–89). The cause is unclear; during the embryonic period, the high hemodynamic stress of such BAVMs could disrupt the spontaneous closure of the PPTA (28). The PPTA can play several roles in BAVMs. First, the PPTA could serve as a path for embolizing the BAVM (Figure 7). Second, for BAVMs, the PPTA could serve as an arterial circulation to maintain hemodynamic balance. Increased hemodynamic stress can promote the enlargement of PPTAs, even those associated with flow-related aneurysms (90). These flow-related aneurysms can be treated following the principles in the aneurysm section in this review.

## Other Roles

PPTA may be associated with other cerebrovascular variants, such as fenestrations and duplications, and rare syndromes, such as PHACE syndrome and Klippel–Feil syndrome (5, 6, 9). In addition, the PPTA may contribute to carotid-basilar collateral flow in the setting of a subclavian steal (91). In VA dissection and BA occlusion, the PPTA may play an important protective role since blood flow to the brainstem is preserved *via* robust collateral flow from the ICA (92, 93). These cerebrovascular variants and disorders are not highly associated with EVT; however, they are not discussed in our review.

## Summary

The PPTA is an important and complex artery of the carotid-basilar connection. It has complex anatomical characteristics. Salas and Saltzman classifications are most often used in PPTA. The PPTA is associated with many vascular disorders, including aneurysms, BAVMs, TCFs, MMD, and large vessel occlusion. A thorough understanding of the anatomical and angiographic features of the PPTA is of utmost importance when making therapeutic decisions for any of these pathological conditions.

## Author Contributions

JY contributed to the conception and design of the manuscript and critically revised the manuscript. JY and YW wrote the manuscript and collected the medical records of the patients. Both authors approved the final version of this manuscript, contributed to the article, and approved the submitted version.

## Conflict of Interest

The authors declare that the research was conducted in the absence of any commercial or financial relationships that could be construed as a potential conflict of interest.

## Publisher's Note

All claims expressed in this article are solely those of the authors and do not necessarily represent those of their affiliated organizations, or those of the publisher, the editors and the reviewers. Any product that may be evaluated in this article, or claim that may be made by its manufacturer, is not guaranteed or endorsed by the publisher.
